# Efectos de la pandemia COVID-19 en la actividad asistencial de los laboratorios clínicos españoles, evolución 2019–2021

**DOI:** 10.1515/almed-2022-0044

**Published:** 2022-10-13

**Authors:** Ana Belén Lasierra Monclús, Álvaro González, Francisco A. Bernabéu Andreu, Imma Caballé Martín, Antonio Buño Soto, Mercè Ibarz, Concepción González Rodríguez, José Puzo Foncillas

**Affiliations:** Servicio de Análisis y Bioquímica Clínica, Hospital Universitario San Jorge, Huesca, España; Servicio de Bioquímica, Clínica Universidad de Navarra, Pamplona, España; Servicio de Bioquímica Análisis Clínicos, Hospital Universitario Puerta de Hierro, Majadahonda, España; Laboratorio Central de CATLAB, Barcelona, España; Servicio de Análisis Clínicos, Hospital Universitario La Paz, Madrid, España; Servicio de Análisis Clínicos, Hospital Universitario Arnau de Vilanova, Lleida, España; Servicio Análisis Clínicos-Bioquímica Clínica, Hospital Universitario Virgen Macarena, Sevilla, España

**Keywords:** actividad asistencial, COVID-19, enfermedades crónicas, laboratorios clínicos, programas poblaciones

## Abstract

**Objetivos:**

Cuantificar el impacto de la pandemia en la actividad asistencial de los laboratorios clínicos españoles.

**Métodos:**

Estudio descriptivo, observacional, retrospectivo y multicéntrico.

**Resultados:**

De marzo a diciembre de 2020 hubo un descenso estadísticamente significativo en el número de solicitudes (−17.7%, p=<0,001) y análisis totales (−18,3%, p<0,001) respecto al mismo periodo de 2019. Se redujo el número de solicitudes de Atención Primaria en un 37,4% (p<0,001) y el número de mediciones de sangre oculta en heces (−45,8%), análisis cualitativo de orina (−30,1%), antígeno prostático específico (PSA) (−28,5%), tirotropina (TSH) (−27,8%), colesterol total (−27,2%) y hemoglobina glicosilada (HbA_1c_) (−24,7%), p<0,001. Se observó un aumento significativo del número de solicitudes de UCI (76,6%, p<0,001) y del número de mediciones de IL-6 (+22,350,9), dímero-D (+617,2%), troponina (+46,8%) y gasometría arterial (+35,9%), p<0,001. Durante los seis primeros meses de 2021, existieron diferencias significativas para análisis cualitativo de orina (−8,7%, p<0,001), PSA (−6,3%, p=0,009), IL-6 (+66.269,2, p<0,001), dímero-D (+603,6%, p<0,001), troponina (+28,7%, p<0,001), gasometría arterial (+26,2%, p=0,014) y ferritina (+16,0%, p=0,002).

**Conclusiones:**

Los laboratorios clínicos españoles han sufrido un cambio en el origen de sus solicitudes y en la demanda de pruebas. Se han incrementado aquellas utilizadas en la evaluación y seguimiento de los pacientes COVID-19, y han disminuido las dirigidas al control de los pacientes no-COVID y a cribados poblacionales. El análisis a más largo plazo refleja una recuperación en las pruebas dirigidas al control de las enfermedades crónicas y se mantiene el aumento del número de mediciones de los biomarcadores utilizados en el manejo de los pacientes COVID-19.

## Introducción

El 31 de diciembre de 2019 se informaron una serie de casos de neumonía de origen desconocido en Wuhan (provincia de Hubei, China) [1]. En enero de 2020, las autoridades sanitarias chinas identificaron un nuevo coronavirus como el agente causal de estos casos de neumonía atípica y publicaron la secuencia genética del virus causante, a este nuevo coronavirus se le llamó 2019-nCoV (del inglés: *2019-novel coronavirus*) [2]. Tras la confirmación oficial por parte de la Organización Mundial de la Salud (OMS) del primer caso registrado fuera de la República Popular China el 13 de enero de 2020, el número de infectados aumentó rápidamente y se comunicaron las primeras muertes. El 11 de febrero de 2020 la OMS anunció que la enfermedad causada por el nuevo coronavirus se denominaría COVID-19, que responde a la forma corta del nombre *coronavirus disease 2019* [1]. El agente causal fue denominado SARS-CoV-2 por el Comité Internacional de Taxonomía de Virus [3]. El 11 de marzo, la OMS caracterizó la enfermedad COVID-19 como pandemia debido a los alarmantes niveles de propagación de la enfermedad, por su gravedad y por la falta de acción. En ese momento, Europa se había convertido en el epicentro de la pandemia, con más casos y muertes notificadas que el resto del mundo junto, al margen de la República Popular China [1]. El primer paciente con COVID-19 registrado en España se diagnosticó el 31 de enero de 2020 en La Gomera (Islas Canarias). El siguiente, el 9 de febrero de 2020 en Palma de Mallorca (Islas Baleares). Ambos casos fueron leves e importados, habían sido contactos de un caso confirmado de SARS-CoV-2 en Alemania y Francia, respectivamente [4]. A finales de febrero de 2020 se registraron los primeros casos en la península, un total de 24 [5], y el 13 de marzo de 2020 el número de casos de COVID-19 probables notificados en España era de 4.209 [6]. El 14 de marzo de 2020 se declaró en España el estado de alarma, implantando el confinamiento domiciliario de la población con el objetivo de frenar la progresión de la pandemia [7]. Debido a estas estrictas medidas, la actividad sanitaria se centró en patologías urgentes, cancelando consultas y cirugías programadas para evitar interacciones y contactos innecesarios [8]. Hay estudios que indican que incluso la actividad urgente de determinadas especialidades también se redujo [9].

El impacto de la pandemia COVID-19 en la asistencia sanitaria ha sido enorme. La actividad de los Servicios, tanto de asistencia primaria como especializada, ha sido crucial en la lucha contra la enfermedad, siendo reconocido el papel de los laboratorios clínicos en el diagnóstico, evaluación y tratamiento de la enfermedad [10]. Los laboratorios clínicos de España tuvieron que adaptarse a estos cambios en la demanda de pruebas dirigidas a establecer la gravedad de estos pacientes, predecir su evolución y realizar una monitorización terapéutica. En las primeras olas, con el rápido aumento de casos e ingresos hospitalarios de enfermos graves, se precisaron numerosas pruebas del laboratorio clínico, incluyendo el rápido desarrollo de nuevos análisis, gestión de escasez de reactivos y suministros, y riesgo de falta de personal debido a la infección por COVID-19.

Por otro lado, el confinamiento domiciliario y la propia pandemia COVID-19 podrían haber causado deficiencias en la atención a otras patologías, como podría ser un menor control de las enfermedades crónicas [11], [12], [13] que se evidenciaría con una disminución de las solicitudes de las pruebas de laboratorio que evalúan dichas enfermedades. Está demostrado que este cambio importante en las actividades diarias ha causado aumento de obesidad y de otros factores asociados a la etiología de las enfermedades crónicas [14], [15], [16].

Con este trabajo se pretende analizar cómo ha cambiado el origen, la complejidad y el tipo de las solicitudes en las pruebas realizadas en los laboratorios clínicos (Servicios de Análisis Clínicos y Bioquímica Clínica) de los hospitales de España. Realizando un estudio de las magnitudes analíticas utilizadas para la evaluación y seguimiento de los pacientes hospitalizados infectados por SARS-CoV-2 y de las pruebas habituales de control realizadas en los laboratorios clínicos a pacientes crónicos como son los diabéticos y oncológicos, entre otros.

El objetivo de este estudio ha sido cuantificar el impacto de la pandemia COVID-19 en la actividad asistencial de los laboratorios clínicos de los hospitales españoles.

## Materiales y métodos

Se diseñó un estudio descriptivo, observacional, retrospectivo y multicéntrico en el que participaron 7 laboratorios de Análisis Clínicos y/o Bioquímica Clínica de diferentes provincias de España. Seis de los laboratorios son de titularidad pública y uno de ellos de titularidad privada (laboratorio 2). Todos ellos atienden pacientes de urgencias, UCI, hospitalizados y consultas externas, siendo el laboratorio 2 el único que no atiende pacientes de Atención Primaria.

Se construyó una base de datos utilizando como registros cada uno de los meses de la serie temporal estudiada (enero de 2019 a junio de 2021). Cada laboratorio recogió de forma retrospectiva y por meses las siguientes variables: número de solicitudes totales y número de solicitudes desglosadas en tipo de origen de solicitud (Urgente, Hospitalizado, UCI, Consultas Externas y Atención Primaria), así como el número de análisis totales y el número de pruebas de glucosa, creatinina, colesterol total, aspartato aminotransferasa (AST), ferritina, troponina, tirotropina (TSH), antígeno prostático específico (PSA), antígeno carcinoembrionario (CEA), sangre oculta en heces (SOH), hemoglobina glicosilada (HbA_1c_), interleucina 6 (IL-6), proteína C reactiva (PCR), análisis cualitativo de orina, gasometría arterial, gasometría venosa y dímero-D. Las pruebas analíticas fueron seleccionadas en función de su relevancia en el manejo del paciente infectado por SARS-CoV-2 [17] y en el control de los pacientes no-COVID (patologías crónicas, programa de cribado de cáncer colorrectal, etc.). No se incluyeron en este estudio las solicitudes ni pruebas del área de Microbiología en aquellos laboratorios clínicos que las realizaban, ya que no son objeto de estudio en este trabajo.

Todos los datos se obtuvieron de los Sistemas Informáticos de Laboratorio (SIL) de cada centro. No se utilizó ningún dato individual de pacientes. Para el estudio se usaron las herramientas de obtención de datos habituales de acuerdo con los procedimientos de trabajo de los laboratorios clínicos, por lo que no se requirió la aprobación del presente trabajo por el Comité de Ética.

### Análisis estadístico

El cálculo de las diferencias absolutas y relativas se realizó tomando como referencia el periodo prepandémico de enero a diciembre de 2019. Las variables no cumplieron criterios de normalidad mediante el test de Kolmogorov-Smirnov, por lo que se analizaron las diferencias estadísticamente significativas con el test de Wilcoxon no paramétrico para datos apareados. Para detectar cambios específicos de cada laboratorio, se aplicó de nuevo el test de Wilcoxon para datos apareados de forma segmentada para cada uno de los laboratorios.

Se calculó el porcentaje de cambio respecto a la media de 2019 de cada una de las variables registradas para cada uno de los laboratorios y se representó gráficamente para analizar de manera objetiva los cambios a lo largo de la serie temporal a estudio.

Todas las pruebas estadísticas se han considerado bilaterales y estadísticamente significativas para valores de p<0,05. Los datos se analizaron con el paquete estadístico SPSS 25.0 (IBM Corporation, Armnok, NY, EEUU).

## Resultados

En la Tabla 1 se muestran los datos globales de los 7 laboratorios participantes, indicando los cambios en el número de solicitudes y número de análisis (diferencias absolutas y diferencias relativas) tras comparar el periodo de marzo a diciembre de 2020 (periodo pandémico) frente al mismo periodo, de marzo a diciembre, de 2019 (periodo prepandémico).

**Tabla 1:** Cambios en la utilización de los laboratorios clínicos (número de solicitudes y número de análisis) entre el periodo pandémico (marzo-diciembre 2020) y el mismo periodo prepandémico más reciente (marzo-diciembre 2019).

	Marzo-Diciembre 2020, n	Marzo-Diciembre 2019, n	Diferencias absolutas, n	Diferencias relativas, %	p-Valor^a^
**Solicitudes totales**	**2.611.129**	**3.173.523**	**−562.394**	**−17,7**	**<0,001**
Solicitudes Atención Primaria	749.444	1.197.783	−448.339	−37,4	<0,001
Solicitudes hospitalización	570.271	551.235	19.036	3,5	0,449
Solicitudes consultas externas	646.752	743.894	−97.142	−13,1	<0,001
Solicitudes urgentes	766.366	805.420	−39.054	−4,9	0,006
Solicitudes UCI	103.814	58.797	45.017	76,6	<0,001
**Análisis totales**	**25.617.642**	**31.357.303**	**−5.739.661**	**−18,3**	**<0,001**
Glucosa	1.608.217	2.061.963	−453.746	−22,0	<0,001
Creatinina	1.432.234	1.782.129	−349.895	−19,6	<0,001
Colesterol total	969.661	1.332.265	−362.604	−27,2	<0,001
AST	1.009.605	1.197.242	−187.637	−15,7	<0,001
Proteína C reactiva	730.604	805.277	−74.673	−9,3	0,107
Ferritina	477.800	513.024	−35.224	−6,9	0,318
IL-6	11.899	53	11.846	22.350,9	<0,001
Troponina	155.194	105.700	49.494	46,8	<0,001
TSH	558.758	773.631	−214.873	−27,8	<0,001
HbA_1c_	274.037	363.784	−89.747	−24,7	<0,001
CEA	63.646	73.014	−9.368	−12,8	0,007
PSA	124.914	174.656	−49.742	−28,5	<0,001
SOH	70.723	130.424	−59.701	−45,8	<0,001
Análisis cualitativo de orina	490.504	701.910	−211.406	−30,1	<0,001
Gasometría arterial	164.800	121.227	43.573	35,9	<0,001
Gasometría venosa	193.295	195.137	−1.842	−0,9	0,560
Dímero-D	170.312	23.748	146.564	617,2	<0,001

^a^Test de Wilcoxon no paramétrico para datos apareados (n=70). AST, aspartato aminotransferasa; CEA, antígeno carcinoembrionario; HbA_1c_, hemoglobina glicosilada; IL-6, interleucina 6; PSA, antígeno prostático específico; SOH, sangre oculta en heces; TSH, tirotropina.

Los resultados de este análisis reflejan una disminución estadísticamente significativa de la actividad asistencial de los laboratorios durante el año 2020 (−17,7% solicitudes totales, p=<0,001 y −18,3% análisis totales, p<0,001) al comparar el periodo de marzo a diciembre de 2020 frente al mismo periodo de 2019. Destaca la disminución del número de solicitudes de Atención Primaria en un 37,4% respecto al mismo periodo de 2019 (p<0,001) así como la reducción en el número de análisis totales en un 18,30%, y en concreto, en el número de mediciones de SOH (−45,8%), análisis cualitativo de orina (−30,1%), PSA (−28,5%), TSH (−27,8%), colesterol total (−27,2%) y HbA_1c_ (−24,7%), p<0,001 para todas ellas.

Por otro lado, el número de solicitudes de UCI durante el año 2020 aumentó de forma significativa respecto al año 2019 (p<0,001), en un 76,6%. Se observa un incremento muy importante en el número de análisis de las pruebas de laboratorio asociadas al manejo de los pacientes COVID-19 como son IL-6 (+22.350,9%, hasta el año 2020 prácticamente inexistente en la gran mayoría de laboratorios), dímero-D (+617,2%), troponina (+46,8%) y gasometría arterial (+35,9%), p<0,001 para todos ellos.

Los datos generales, a nivel de todos los laboratorios participantes, muestran una disminución mucho más significativa en el número de solicitudes totales cuando se analiza la primera ola de la pandemia (de marzo a mayo de 2020) respecto al mismo periodo de 2019 (−35,7%, p<0,001), destacando un descenso del número de solicitudes de Atención Primaria (−63,9%, p<0,001) y de consultas externas (−38,3%, p<0,001), así como un aumento del número de solicitudes de UCI (+100,5%, p=0,001). Durante este periodo, el número de análisis totales descendió un 40,6%, p<0,001.

Para valorar la repercusión de la pandemia COVID-19 a más largo plazo también se analizó la actividad asistencial de los laboratorios durante el periodo de enero a junio de 2021 y se comparó con los datos del mismo periodo del año 2019 (Tabla 2).

**Tabla 2:** Cambios, a más largo plazo tras el inicio de la pandemia, en la utilización de los laboratorios clínicos (número de solicitudes y número de análisis) comparando el periodo pandémico (enero a junio de 2021) frente al mismo periodo prepandémico más reciente (enero a junio de 2019).

	Enero-Junio 2021, n	Enero-Junio 2019, n	Diferencias absolutas, n	Diferencias relativas, %	p-Valor^a^
**Solicitudes totales**	**2.001.270**	**1.965.293**	**35.977**	**1,8**	**0,126**
Solicitudes Atención Primaria	677.047	745.478	−68.431	−9,2	<0,001
Solicitudes hospitalización	426.161	342.390	83.771	24,5	0,002
Solicitudes consultas externas	483.598	462.865	20.733	4,5	0,123
Solicitudes urgentes	501.461	488.654	12.807	2,6	0,778
Solicitudes UCI	51.392	35.084	16.308	46,5	<0,001
**Análisis totales**	**19.797.233**	**19.615.906**	**181.327**	**0,9**	**0,330**
Glucosa	1.246.609	1.289.491	−42.882	−3,3	0,232
Creatinina	1.103.286	1.112.368	−9.082	−0,8	0,817
Colesterol total	791.416	840.895	−49.479	−5,9	0,062
AST	765.502	763.097	2.405	0,3	0,253
Proteína C reactiva	519.752	511.334	8.418	1,7	0,022
Ferritina	378.728	326.457	52.271	16,0	0,002
IL-6	8.628	13	8.615	66.269,2	<0,001
Troponina	86.352	67.090	19.262	28,7	<0,001
TSH	488.524	485.763	2.761	0,6	0,232
HbA_1c_	246.167	224.906	21.261	9,5	<0,001
CEA	46.118	45.837	281	0.6	0,604
PSA	105.350	112.400	−7.050	−6,3	0,009
SOH	79.120	81.893	−2.773	−3,4	0,167
Análisis cualitativo de orina	394.364	431.893	−37.529	−8,7	<0,001
Gasometría arterial	105.844	83.890	21.954	26,2	0,014
Gasometría venosa	132.314	115.937	16.377	14,1	<0,001
Dímero-D	103.844	14.760	89.084	603,6	<0,001

^a^Test de Wilcoxon no paramétrico para datos apareados (n=42). AST, aspartato aminotransferasa; CEA, antígeno carcinoembrionario; HbA_1c_, hemoglobina glicosilada; IL-6, interleucina 6; PSA, antígeno prostático específico; SOH, sangre oculta en heces; TSH, tirotropina.

Se observa que durante dicho periodo de 2021 se alcanzaron cifras de número total de solicitudes similares a las de 2019 (+1,8%, p=0,126), aunque el origen de dichas solicitudes fue diferente, con un aumento respecto a 2019 en el número de solicitudes de UCI (+46,5%, p<0,001) y en el número de solicitudes de hospitalizados (+24,5%, p=0,002) y un descenso en el número de solicitudes de Atención Primaria (−9,2%, p<0,001), menos acusado que en 2020. De forma similar, el número de análisis totales realizados en 2021 se recuperó respecto a 2020 y alcanzó cifras de 2019 (+0,9%, p=0,330). El número de mediciones de SOH (−3,4%), análisis cualitativo de orina (−8,7%) y PSA (−6,3%) todavía no habían alcanzado valores de 2019, pero habían aumentado respecto a 2020. Por su parte, en 2021 habían aumentado los análisis de HbA_1c_ respecto a 2019 (+9,5%, p<0,001).

Por otro lado, la tendencia al alza de la solicitud de magnitudes asociadas a la COVID-19 se mantuvo durante el año 2021. Como muestran los resultados, con un incremento respecto a 2019 de número de análisis de IL-6 (+66.269,2, p<0,001), dímero-D (+603,6%, p<0,001), troponina (+28,7%, p<0,001), gasometría arterial (+26,2%, p=0,014) y ferritina (+16,0%, p=0,002), aumentos que son incluso mayores que los observados en 2020 para IL-6 y ferritina.

**Figura 1:**
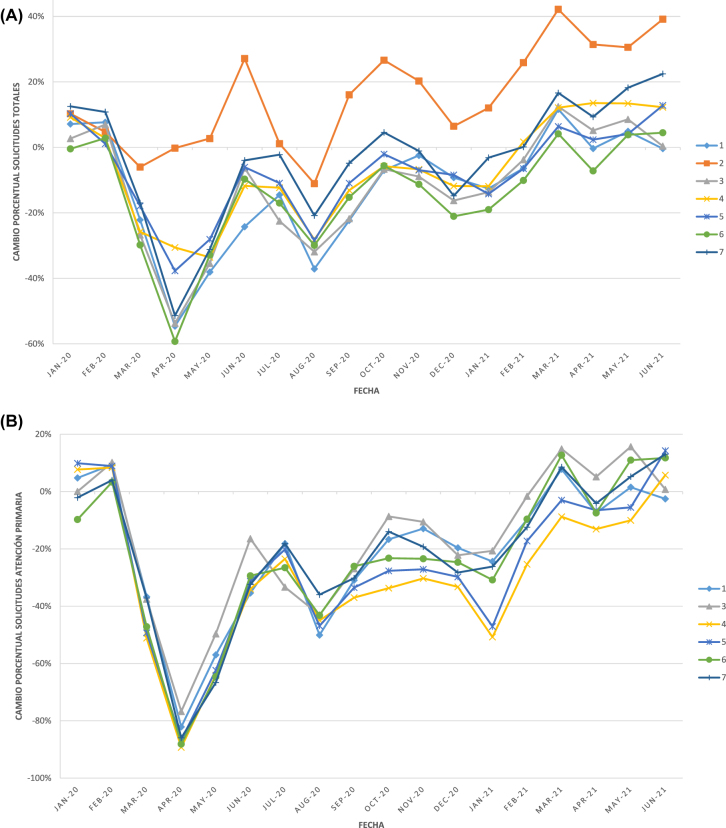

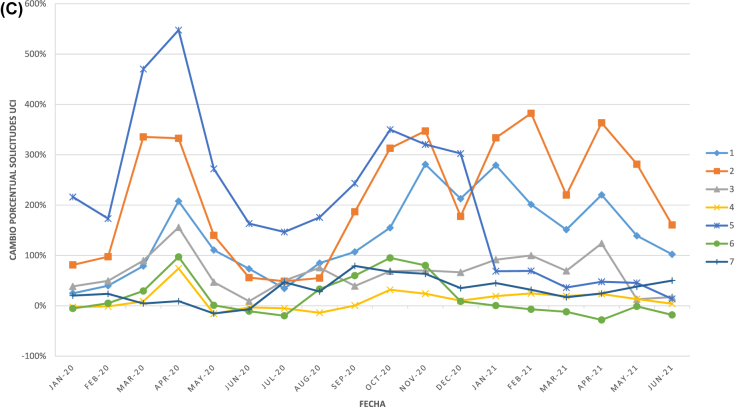
Cambio porcentual en el número de solicitudes de cada uno de los 7 laboratorios, respecto a su correspondiente media de 2019, a lo largo de la serie temporal estudiada. (A) Número solicitudes totales; (B) Número solicitudes Atención Primaria (el laboratorio 2 no atiende este tipo de pacientes por eso no aparece en la gráfica); (C) Número solicitudes UCI.

**Figura 2:**
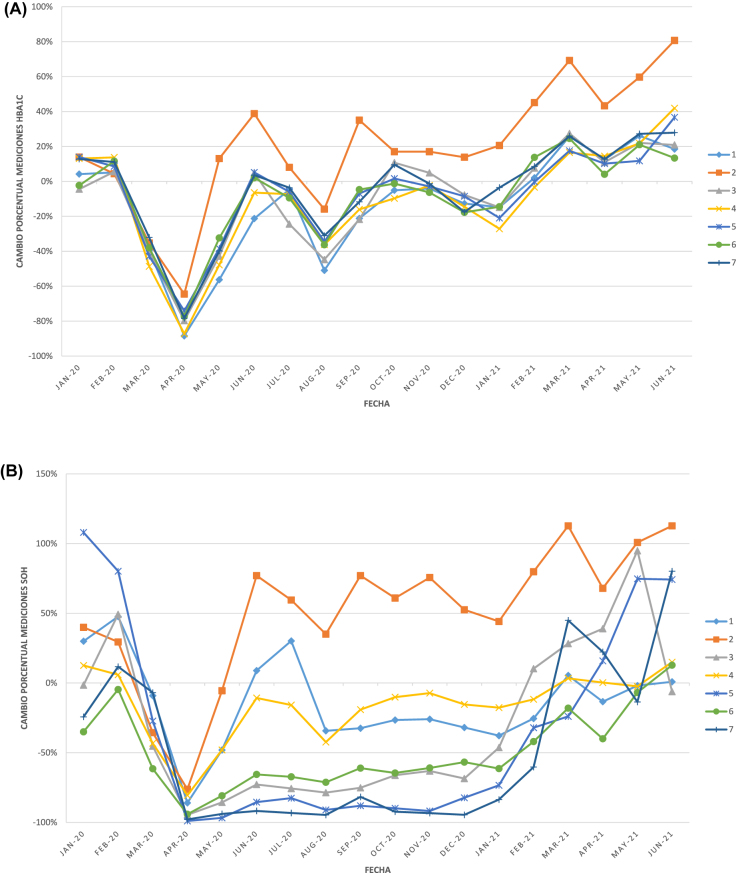

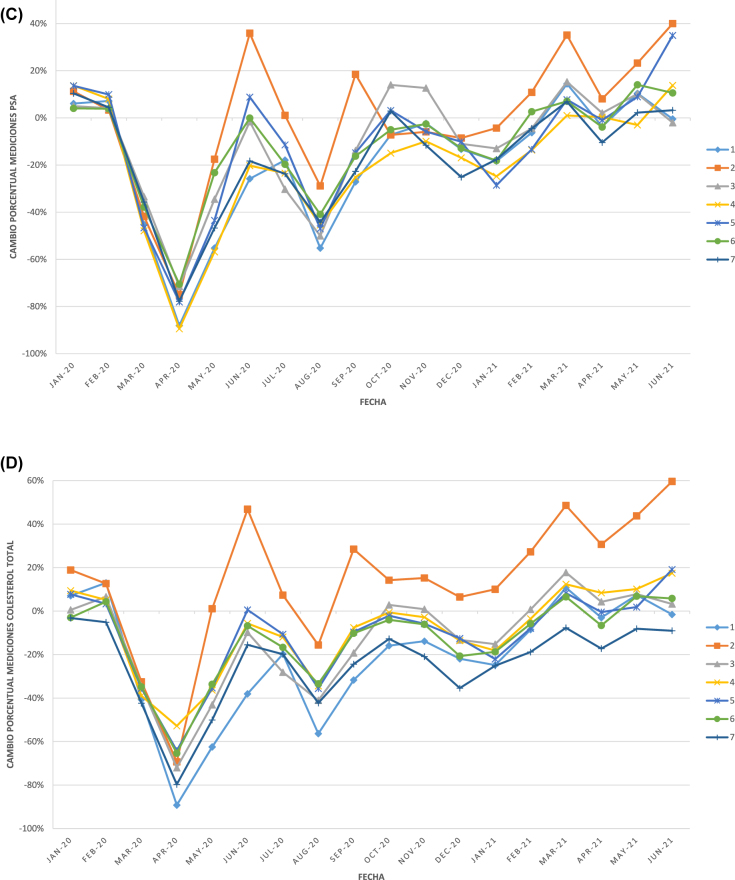
Cambio porcentual en el nº de pruebas de laboratorio asociadas al control de pacientes no-COVID de cada uno de los 7 laboratorios, respecto a su correspondiente media de 2019, a lo largo de la serie temporal estudiada. (A) HbA_1c_; (B) SOH; (C) PSA; (D) colesterol total. HbA_1c_, hemoglobina glicosilada; PSA, antígeno prostático específico; SOH, sangre oculta en heces.

En los siguientes gráficos se pueden observar los porcentajes de cambio del número de solicitudes respecto a la media de 2019 de cada uno de los 7 laboratorios desde enero de 2020 a junio de 2021 (Figura 1), así como los cambios porcentuales del número de análisis de varios de los analitos estudiados (Figuras 2 y 3).

**Figura 3:**
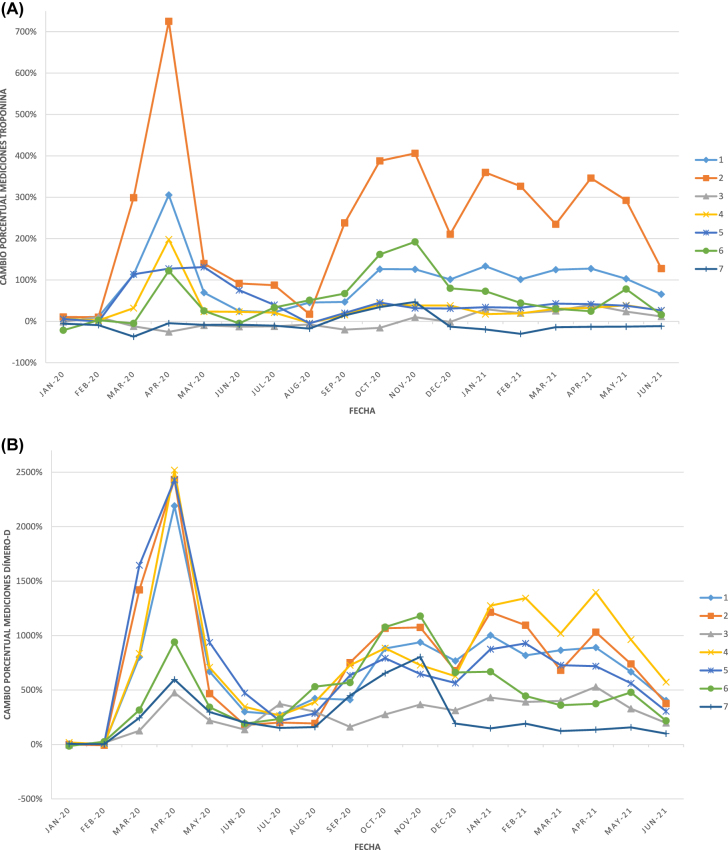

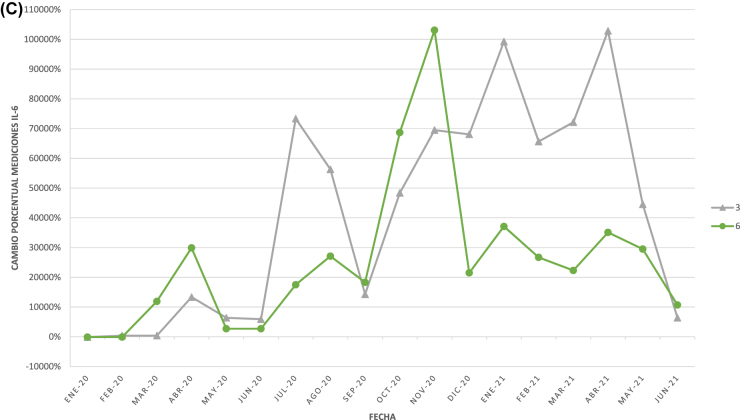
Cambio porcentual en el nº de pruebas de laboratorio asociadas al control y manejo de pacientes COVID-19 de cada uno de los 7 laboratorios, respecto a su correspondiente media de 2019, a lo largo de la serie temporal estudiada. (A) Troponina; (B) Dímero-D; (C) IL-6 (sólo se muestran los datos de los laboratorios que disponían de la técnica en 2019). IL-6, interleucina 6.

## Discusión

En marzo de 2020, a causa de la emergente pandemia COVID-19, numerosos países declararon el estado de alarma en un intento de controlar la propagación del virus y evitar el colapso de los sistemas de salud. España fue uno de los países más afectados por la aplicación de estas medidas, que incluía el confinamiento domiciliario de ciudadanos y la reducción de la actividad laboral a los servicios esenciales.

Estudiar los cambios en la actividad asistencial de los laboratorios clínicos de España ocurridos durante la pandemia es necesario para poder planificar estrategias frente a futuras pandemias y gestionar los recursos sanitarios adecuadamente.

La mayoría de los estudios existentes sobre la utilización del laboratorio durante la pandemia COVID-19 se han centrado principalmente en el diagnóstico del SARS-CoV-2, siendo esenciales para ello los laboratorios de Microbiología [18]. También se han realizado evaluaciones de pruebas para el diagnóstico, la monitorización de la gravedad y el pronóstico de los pacientes infectados por SARS-CoV-2 [19, 20]. Sin embargo, son escasos los artículos publicados actualmente que evalúen la demanda de recursos COVID y no COVID-19 en los laboratorios clínicos [12, 13], casi ninguno de ellos ha valorado el efecto de la pandemia sobre magnitudes asociadas al control de otras enfermedades crónicas [21], solicitadas principalmente desde Atención Primaria, y ninguno de ellos ha sido realizado en nuestro país.

Durant et al. [12] observaron un descenso en el volumen de pruebas (−27,6%) en su laboratorio central durante la segunda y tercera semana de marzo de 2020 respecto al mismo periodo de 2019, siendo el resultado menor que el detectado en nuestro estudio durante la primera ola de la pandemia (de marzo a mayo de 2020) respecto al mismo periodo de 2019 (−40,6% análisis totales). También informaron un aumento en las mediciones de ferritina (+335,6%), troponina (+235,6%), dímero-D (+1.771,6%) y gasometría arterial (+24,1%), siendo nuestros resultados: troponina (+46,8%), dímero-D (+617,2%) y gasometría arterial (+35,9%), mientras que no observamos diferencias significativas para la ferritina (−6,9%, p=0,318). Aunque el número de solicitudes de ferritina ha aumentado durante la pandemia por su asociación con el estado inflamatorio del paciente infectado por SARS-CoV-2, este hecho no se ve reflejado en nuestros resultados probablemente debido a que los estudios de anemia se vieron reducidos considerablemente. Duran et al. [12] sólo llevaron a cabo un análisis descriptivo de los datos, no realizaron un estudio estadístico para valorar de forma objetiva los cambios en el volumen de pruebas y tampoco analizaron otras magnitudes asociadas al manejo de pacientes no COVID-19.

Durante la pandemia, la atención a personas con afecciones crónicas disminuyó debido a la restricción de las visitas de atención médica electivas y no urgentes y un mayor temor sobre la posible exposición al coronavirus durante las visitas presenciales a los centros asistenciales [11]. Las enfermedades crónicas son la causa de muerte de 41 millones de personas cada año, lo que equivale al 71% de todas las muertes a nivel mundial. Las enfermedades cardiovasculares (17,9 millones de personas al año), los cánceres (9,3 millones), las enfermedades respiratorias (4,1 millones) y la diabetes (1,5 millones) suponen el 80% de las muertes prematuras [22]. En Estados Unidos se analizó el impacto de la COVID-19 en seis afecciones crónicas (insuficiencia cardiaca congestiva, enfermedad pulmonar obstructiva crónica, diabetes tipo 2, hipertensión, enfermedad renal crónica y cáncer), con reducciones del 50% en nuevos diagnósticos de todas ellas y reducciones en las visitas médicas entre un 30 y un 60%. A finales de junio de 2020, las visitas se recuperaron hasta llegar an un 70–85% de los datos de años anteriores, mientras que las visitas no médicas (que incluían las pruebas de imagen y laboratorio, entre otras) se recuperaron sólo hasta un nivel del 55% [13]. Recuperación menor que la observada en nuestro estudio, con un porcentaje de solicitudes totales del 79,1% en junio de 2020 respecto al mismo periodo de 2019, aunque mayor si tenemos en cuenta únicamente las solicitudes de Atención Primaria (29,7% en nuestro caso).

En España, durante la primera ola, Atención Primaria redujo drásticamente la actividad programada (incluyendo las pruebas analíticas, el seguimiento a pacientes crónicos y los programas poblacionales de cribado de cáncer) al verse orientados a priorizar la atención COVID-19 [23]. Hemos observado una disminución del número de solicitudes de Atención Primaria al comparar el periodo de marzo a diciembre de 2020 frente al mismo periodo de 2019, y esta reducción fue mayor durante la primera ola (−63,9%). Resultado similar al publicado por Nagy et al. [21], que varió entre un −46% y un −71% para los autoanticuerpos solicitados habitualmente desde Atención Primaria. Nuestros datos muestran una disminución en el número de mediciones de SOH, análisis cualitativo de orina, PSA, TSH, colesterol total y HbA_1c_ durante el periodo de marzo a diciembre de 2020 respecto al mismo periodo de 2019. Lo que evidencia una reducción significativa durante el año 2020 del seguimiento y control de los pacientes no-COVID (diabetes, enfermedades tiroideas, dislipemias, etc.), así como del cribado poblacional de cáncer colorrectal llevado a cabo desde Atención Primaria.

Realizamos un seguimiento a más largo plazo (hasta junio de 2021), para valorar la situación de los laboratorios clínicos más de un año después del inicio de la pandemia. Aunque el número de solicitudes de Atención Primaria durante los primeros seis meses de 2021 fue significativamente inferior al mismo periodo de 2019 (−9,2%, p<0,001), para la mayoría de magnitudes ya no se observaron diferencias significativas respecto a 2019 (Tabla 2). Estos resultados sugieren que en 2021 se estaba volviendo a recuperar el nivel de control de las patologías crónicas que durante el año 2020 se había visto disminuido de forma muy significativa, y todavía más acusado durante la primera ola de la pandemia. Para la mayoría de magnitudes esta recuperación ha sido progresiva y muy similar en los distintos laboratorios (Figura 2). Sin embargo, destaca el descenso drástico de la prueba de SOH desde el inicio de la pandemia (prácticamente −100%), que se mantuvo así durante todo el año 2020 en varios de los hospitales estudiados y cuya recuperación ha sido muy diferente en función del laboratorio analizado (Figura 2C). Estos datos indican que el cribado de cáncer colorrectal ha sido retomado más tarde en unas provincias o áreas de salud que en otras, con la correspondiente pérdida de casos captados durante todo el año 2020.

Por otro lado, nuestros resultados muestran un aumento en la solicitud de los biomarcadores utilizados en la evaluación y seguimiento de los pacientes hospitalizados infectados por SARS-CoV-2 (IL-6, dímero-D, troponina, gasometría arterial y ferritina), que incluso se habían incrementado en 2021 respecto a 2020 para IL-6 y ferritina. Desde el inicio de la pandemia el conocimiento sobre la IL-6 en el manejo y tratamiento de los pacientes infectados por SARS-CoV-2 fue aumentando progresivamente [17, 24], [25], [26], [27], y su uso se fue estandarizado en los laboratorios clínicos a lo largo del año 2020 como marcador precoz de respuesta inflamatoria grave, predictor de gravedad [17, 24, 25] y como criterio para el inicio de tratamiento con inhibidores de IL-6 [26, 27]. De los 7 laboratorios participantes, únicamente 3 disponían de la prueba antes de la pandemia y el resto fueron incorporando dicho marcador a sus carteras de servicio a lo largo del año 2020. En cuanto a la ferritina, prueba de laboratorio utilizada tanto en los estudios básicos de anemia como en el seguimiento de los pacientes COVID-19, nuestro estudio no detectó un aumento significativo de su solicitud durante 2020 probablemente debido a que los estudios de anemia se vieron reducidos considerablemente, pero sí en 2021 al haberse recuperado el número de solicitudes y pruebas dirigidas al control de pacientes no-COVID y continuar aumentadas las solicitudes de pacientes infectados por SARS-CoV-2 hospitalizados y de UCI.

El análisis gráfico de la evolución de la actividad asistencial de los laboratorios de Análisis Clínicos y/o Bioquímica Clínica a lo largo de la serie temporal de los 30 meses estudiados (Figuras 1uras –Figuras 3), muestra que tanto el número de solicitudes como el número de análisis llevados a cabo durante dicho periodo siguen gráficamente las distintas olas de la COVID-19 (primera ola: marzo-junio 2020; segunda ola: julio-diciembre 2020; tercera ola: diciembre 2020-marzo 2021; cuarta ola: marzo-junio 2021 [28]). Observándose los mismos picos en el número de solicitudes de UCI (Figura 1C) y de magnitudes asociadas al control de los pacientes infectados por SARS-CoV-2 (Figura 3), mientras que se observan picos inversos tanto en el número de solicitudes totales como de Atención Primaria (Figura 1A, B) y en el número de análisis de las magnitudes utilizadas en el manejo de los pacientes no-COVID (Figura 2). Se observan diferencias en el impacto que las distintas olas han tenido en la demanda de cada uno de los laboratorios clínicos, hecho que podría explicarse por las diferencias de presentación de la pandemia en los distintos territorios, así como por factores de organización sanitaria no analizados en este estudio (Figura 1C y 3).

Como muestran los datos de este trabajo, durante el año 2020 los laboratorios de Análisis Clínicos y/o Bioquímica Clínica de España han sufrido un cambio drástico en el origen de sus solicitudes y en la demanda de pruebas por parte de los clínicos. Los laboratorios han tenido que adaptarse rápidamente a estos cambios, reorganizar sus recursos, crear perfiles específicos para pacientes COVID-19 e incorporar nuevas pruebas analíticas (como IL-6) [17, 24, 25, 29]. En hipotéticas situaciones de crisis sanitarias estos hallazgos pueden servir para gestionar y distribuir los recursos de forma más adecuada. Se deben tener en cuenta recursos materiales y personales, dentro de los laboratorios clínicos, de un hospital o incluso entre laboratorios de distintos territorios. Los laboratorios clínicos deben anticiparse y coordinarse para responder rápidamente a estas demandas cambiantes. Nuestros resultados aportan una descripción general de lo ocurrido que puede ayudar a la toma de decisiones operativas.

Entre las limitaciones de este estudio destacamos las siguientes. En primer lugar, a pesar de tratarse de un estudio multicéntrico con la participación de 7 laboratorios de diferentes provincias, puede no representar a todo el territorio español. En segundo lugar, no se han estudiado factores de organización sanitaria que pudieran introducir sesgos en el análisis de los resultados. Y por último, no se han analizado diferencias entre laboratorios, aunque sí se ha comprobado estadísticamente que los resultados obtenidos son representativos de los 7 laboratorios participantes.

En conclusión, los laboratorios clínicos españoles han ido adaptando su trabajo a los requerimientos y necesidades de las distintas olas de la pandemia, siendo este el primer estudio que analiza estos datos en España y que además valora la demanda de pruebas analíticas utilizadas en el control y manejo de patologías no-COVID. Las solicitudes de magnitudes utilizadas en la evaluación y seguimiento de los pacientes COVID-19 hospitalizados aumentaron de forma muy rápida desde marzo de 2020. Por otro lado, se ha confirmado la disminución de las solicitudes de pruebas analíticas dirigidas al control de las enfermedades crónicas más prevalentes. Como se ha observado desde el principio de la pandemia, la diabetes, la hipertensión o la obesidad constituyen factores de riesgo que aumentan la morbimortalidad de la COVID-19 [15, 16, 30]. En los próximos años veremos la repercusión que ha tenido la disminución del control y manejo de las patologías crónicas y la reducción de los programas de cribado durante el año 2020, y su impacto en la morbimortalidad y en la futura demanda de los servicios sanitarios a los laboratorios clínicos.
